# Are Optometrists Prepared to Be Involved in Post-Stroke Rehabilitation?

**DOI:** 10.3390/diagnostics14202307

**Published:** 2024-10-17

**Authors:** Amritha Stalin, Susan J. Leat, Tammy Labreche

**Affiliations:** 1 School of Optometry & Vision Science, University of Waterloo, Waterloo, ON N2L 3G1, Canada; susan.leat@uwaterloo.ca (S.J.L.); tammy.labreche@uwaterloo.ca (T.L.); 2 Centre for Eye and Vision Research (CEVR), 17W, Hong Kong Science Park, Hong Kong

**Keywords:** optometrists, stroke, vision care, vision rehabilitation

## Abstract

Background/Objectives: Stroke survivors often experience various visual consequences that impact their daily life and may benefit from visual interventions. However, some of these usually go unaddressed as optometrists are rarely included in the post-stroke care pathway. Yet, optometrists are interested in contributing to the care of these patients. This survey evaluated the readiness of optometrists in diagnosing and managing visual disorders specific to stroke survivors. Methods: A questionnaire was developed by the researchers, pilot tested by 5 research optometrists and 15 community optometrists, and modified based on the feedback. Practicing optometrists were invited to complete the anonymous online survey through optometric organizations in Canada, the US, Hong Kong, India, and the UK. Results: Most respondents displayed strong knowledge, but 61.6% indicated that enhancing their knowledge would be helpful. The majority (87%) agreed that stroke is related to an increased incidence of falls. Participants’ knowledge regarding the natural history of post-stroke visual disorders was poorer. There were also inconsistencies regarding what optometrists considered ideal interventions and what they undertook in practice. More than 50% of respondents reported that the quality of published evidence on post-stroke visual consequences was low or nonexistent. Conclusions: Overall, survey respondents displayed sufficient knowledge. However, there are areas of uncertainty in their knowledge, which in many cases correspond to real gaps in the available evidence. There is a need to identify and remediate these gaps to enable optometrists to deliver quality optometric care as collaborative members of the post-stroke professional team, which would eventually improve the rehabilitation of stroke survivors.

## 1. Introduction

A stroke can be defined as neurological damage caused by the occlusion or hemorrhage of blood vessels that supply the brain, and can result in motor and sensory losses [1]. The definition of stroke now includes “ocular stroke”, caused by occlusion of one of the retinal arteries [2]. Because a significant portion of the central nervous system is allocated to processing visual information, a stroke can cause a variety of visual deficits which can include deficits in visual field [3], visual acuity [4], color vision [5], or depth perception [6,7]. Strokes affecting the brainstem or the efferent pathways may result in eye movement disorders, such as nystagmus, ocular motor apraxia, gaze palsies, extra-ocular muscle paresis, or palsy resulting in strabismus or double vision [3,4,8,9,10,11,12]. Similarly, a wide range of visual perceptual problems can occur post-stroke depending on the area of the brain and the visual pathway affected. These include agnosia, visual neglect, visual midline shift, visual–spatial deficits, prosopagnosia, simultanagnosia, cerebral achromatopsia, and optic ataxia [6,7]. It is estimated that approximately 60% of stroke survivors have some visual, eye movement, or visual perceptual disorder (excluding pre-existent eye problems) [13].

These visual deficits may cause a wide range of visual disabilities such as difficulty in judging distances, reaching for objects, navigating the environment safely, reading, and driving, as well as issues with hand–eye coordination, balance, and mobility [14]. They can also lead to falls or accidents [10,11,12]. If not addressed, these visual problems can negatively impact a person’s daily activities, rehabilitation process, and quality of life [3,6,7,10,15,16].

Current stroke rehabilitation focusses on the recovery of physical and cognitive abilities and typically involves a team of healthcare professionals including physical therapists, occupational therapists, speech therapists, and others, but not optometrists [17,18,19]. The visual or visual perceptual consequences of stroke may impact an individual’s performance during rehabilitation activities and may impede the recovery process [20]. The alleviation of visual symptoms and disorders is also important for the direct benefits that can be attained. Therefore, early vision assessment and the management of visual problems are crucial parts of post-stroke rehabilitation, and including vision care practitioners in the stroke care team would be beneficial.

Although the diagnosis and management of post-stroke visual consequences is important in the recovery process for individuals post-stroke, optometrists are rarely included in the stroke care team [17,21], with the result that in-depth visual evaluations not being performed on most patients post-stroke [19]. However, according to an international survey regarding the optometric management of stroke survivors, optometrists are interested in being more involved in the stroke care team [19]. The current study provides an analysis of optometrists’ preparedness to diagnose and manage the visual and visual perceptual symptoms that arise post-stroke. While stroke rehabilitation teams typically focus on physical and cognitive recovery, the visual deficits experienced by stroke survivors often go underdiagnosed and untreated, despite their significant impact on rehabilitation outcomes. This study examines the readiness of optometrists to fill this gap, highlighting the need for further integration of vision care into post-stroke rehabilitation pathways. This is important because it will help identify where additional training is required and where the involvement of optometrists in multidisciplinary stroke care teams could be enhanced. The results of this study will be valuable to healthcare planners, policy makers, and clinicians, as they identify actionable insights that can be used to design more inclusive and effective post-stroke rehabilitation programs.

## 2. Materials and Methods

The cross-sectional survey was conducted in compliance with the Declaration of Helsinki and received ethical clearance from multiple institutions. Ethics clearance was granted by the University of Waterloo Research Ethics Board at the School of Optometry and Vision Sciences, the Research Ethics Committee at Cardiff University, and the ethics sub-committee at the Vision Research Foundation (India). Additional ethical approval was not required for conducting this online survey in Hong Kong and the US.

The researchers (all optometrists) developed and tested a questionnaire based on their clinical expertise and knowledge of stroke literature. For details of the development of the questionnaire, and the full questionnaire, see Stalin et al. [19]. Briefly, the questionnaire was evaluated both by other researchers in the field and by optometrists practicing in each country, who gave feedback regarding the clarity, lack of ambiguity, and appropriateness of the questions for their country. The questionnaire was modified based on this feedback. In order to include Canadian Francophones, the survey was translated into French. However, it was not necessary to translate the survey for the other countries, as it was reported that all optometrists were proficient in English. The French version of the questionnaire was reviewed by a practicing Francophone optometrist to ensure that the translation preserved the meaning and clarity of the original English version (this optometrist was also one of those who reviewed the questions for clarity, etc., and practices in both English and French). Additionally, one of the researchers back-translated the questions using an online translation service (Google Translate) to verify accuracy. The anonymous online REDCap survey was available from June 2021 to December 2021. Practicing optometrists were invited through their respective associations (see Acknowledgements).

Two hundred and seventy-six optometrists (Canada—45, Hong Kong—30, India—34, UK—30, and US—136) responded to the invitation to participate in the survey. One respondent chose not to indicate their country of practice and so their data could not be included in the country-wise analysis. Data were exported from REDCap into Excel (Microsoft^®^ Excel^®^ for Microsoft 365 MSO, Version 2112), which was used for descriptive data analysis, and Jamovi (version 2.3.18.0) was used for country-specific comparisons of age, gender, and number of practicing hours (Kruskal–Wallis one-way analysis of variance). The Shapiro–Wilk test was used to assess normality. Confidence intervals for gender proportions were calculated using the Clopper–Pearson method.

## 3. Results

The ages of the participants ranged from 21 to 79 years, with 61% participation from females and 37% from males, and the majority had been practicing for more than 10 years. The remaining 2% chose not to answer the question regarding their gender. At the time of the study, the estimated number of registered optometrists was 5000 in Canada [22], 2000 in Hong Kong [23], 9000 in India [24], 14,000 in the UK [25], and 46,000 in the US [26]. The demographics and practicing details of the participant population according to each country were reported by Stalin et al. [19].

The age distribution of the respondents was similar across countries, averaging between 44 and 49 years, with the exception of India, where the average was 31.5 years (*p* < 0.01). More females responded from Canada (82%) compared to Hong Kong (57%), the UK (47%), and the US (60%). In India, 71% of the respondents were female. The weekly practicing hours of participants ranged from 4 to 72 and this was similar between countries (*p* > 0.05).

In terms of experience, 34% of the respondents had more than 25 years of experience as a qualified optometrist, 37% had 0–10 years, and 29% had 11–25 years. Most worked in solo/independent practices (25%), followed by educational institutions (19%) and hospitals (17%). Additionally, 94% of Indian respondents, 85% from the US, 73% from Hong Kong, 67% from the UK, and 53% from Canada reported a specialized area of expertise within optometry, such as low vision therapy, ocular disease, or myopia management.

**Figure 1:**
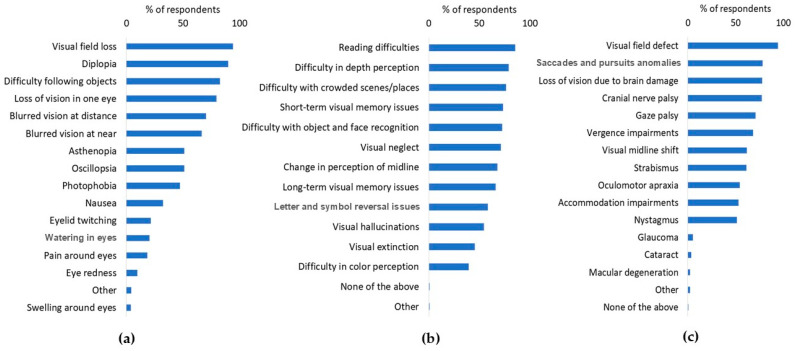
Survey results regarding knowledge of post-stroke across all countries, in response to the question “Which of the following could be experienced by an individual post-stroke (due to the stroke)”. (**a**) Visual symptoms, (**b**) visual perceptual difficulties, and (**c**) visual disorders.

**Figure A1:**
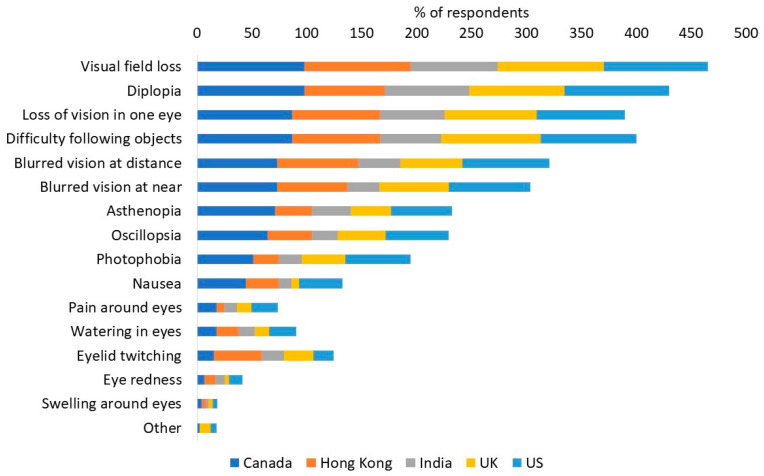
Country-specific percentages of respondents regarding visual symptoms.

Respondents were asked about a range of visual symptoms, visual perceptual difficulties, and disorders that often occur post-stroke. The questionnaire also included some “distractors”, i.e., disorders that are not typically associated with stroke (such as eye redness or pain around the eyes), so that participants would be challenged to consider which were or were not associated with stroke. Over 65% of the respondents demonstrated familiarity with many common visual symptoms that are known to occur post-stroke, including visual field loss, diplopia, eye movement-related problems, and blurred vision. The majority (about 80%) of participants were not acquainted with less common symptoms such as asthenopia, oscillopsia, photophobia, nausea, and eyelid twitching [27]. A few participants (4%) were familiar with additional symptoms that are associated with stroke, such as ptosis and lagophthalmos (see Figure 1a). Some participants (20% or less) incorrectly associated epiphora, pain around the eyes, redness, and swelling with stroke. These findings were comparable between participants from Canada, the UK, and the US. While more than 80% of the participants from India and Hong Kong were knowledgeable about common symptoms such as visual field defects and diplopia, the percentage of respondents from these countries who were aware of other visual symptoms was lower compared with those of other countries (see Appendix A, Figure A1).

**Figure A2:**
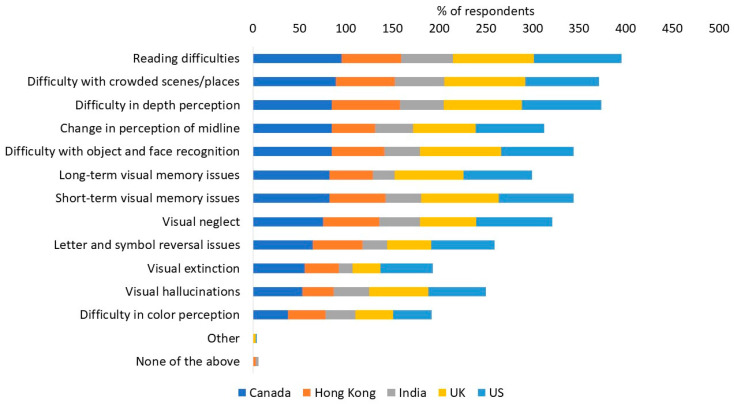
Country-specific percentages of respondents regarding visual perceptual symptoms.

Similarly, over 65% of the respondents were knowledgeable about many visual perceptual difficulties, including reading difficulties, depth perception, recognition of objects and faces, perception of visual midline, visual neglect, and short- and long-term visual memory issues. However, most respondents were less aware of visual extinction and color perception difficulties being related to stroke (see Figure 1b). The outcomes were comparable among participants from Canada, the UK, and the US, although respondents from India and Hong Kong showed less awareness of visual perceptual difficulties. (Appendix A, Figure A2).

**Figure A3:**
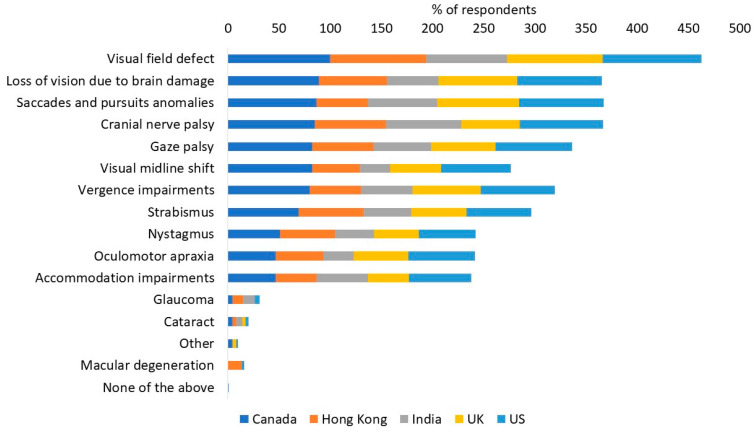
Country-specific percentages of respondents regarding visual disorders.

Over 65% of the participants were aware of post-stroke vision disorders such as vergence impairments, gaze palsies, cranial nerve palsies, loss of vision, saccade and pursuit abnormalities, and visual field defects. Conversely, disorders like nystagmus, accommodation impairments, oculomotor apraxia, strabismus, visual midline shifts, and macular degeneration were less recognized (Figure 1c). A few participants (5% or less) incorrectly associated glaucoma and cataract with stroke. These findings were consistent among participants from all countries except India, where a lower percentage of respondents recognized all visual disorders (Appendix A, Figure A3).

**Figure 2:**
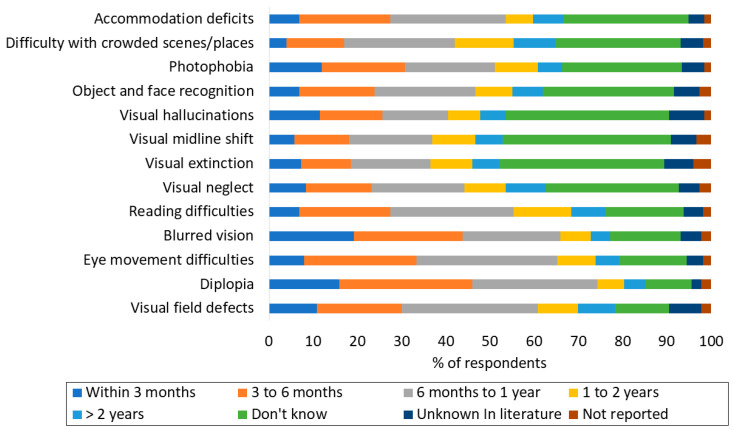
The survey results on the knowledge of the natural history of post-stroke visual problems. The percent responses are in answer to the question “If post-stroke patients are going to recover, either partially or completely, during what maximum time period do you expect their recovery from each of the following to occur?”.

While most respondents reported that they are aware of the natural progression of visual symptoms like reading difficulties, blurred vision, eye movement problems, diplopia, and visual field defects, many (40% or less) were unfamiliar with the natural progression of symptoms and disorders such as accommodation deficits, difficulty in crowded places, photophobia, recognition of objects and faces, visual hallucinations, visual midline shift, visual extinction, and visual neglect (Figure 2).

**Figure A4:**
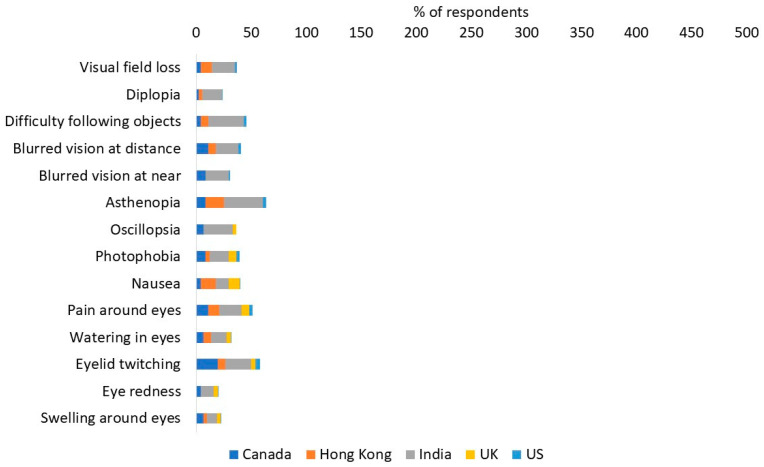
Country-specific percentages of respondents regarding visual problems that may be psychosomatic.

Out of all the respondents, 77% thought that the majority of visual symptoms experienced post-stroke are not psychosomatic. This was consistent across all countries except for India, where only 29% thought this (Appendix A, Figure A4).

**Figure A5:**
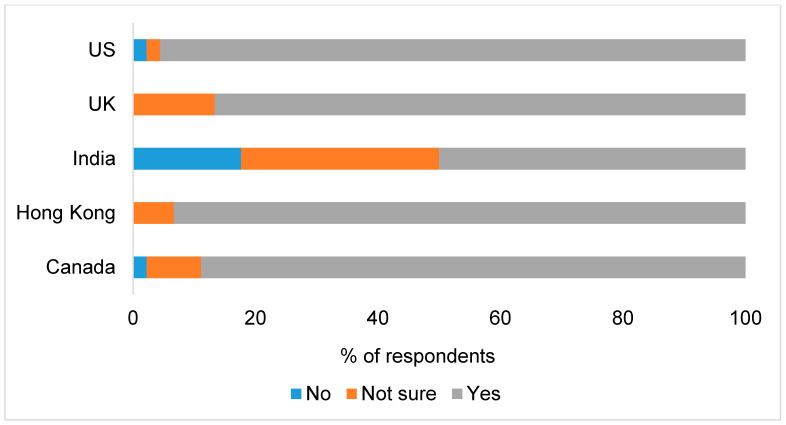
Country-specific percent responses in answer to the question “Is stroke related to increased incidence of falls?”.

Overall, 87% of the respondents agreed that stroke is associated with a higher incidence of falls, while 9% were uncertain, and 4% stated that falls are not related to stroke. This was similar across all countries, except in India, where 32% were uncertain and 18% believed that stroke is not related to an increased incidence of falls (Appendix A, Figure A5).

**Figure 3:**
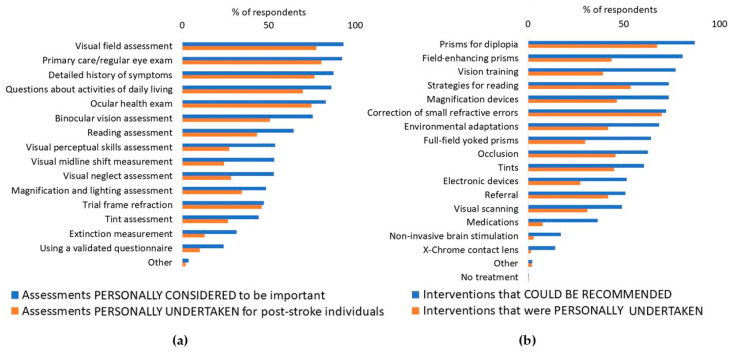
The survey results regarding (**a**) the visual assessments and (**b**) interventions for an individual after a stroke.

**Figure A6:**
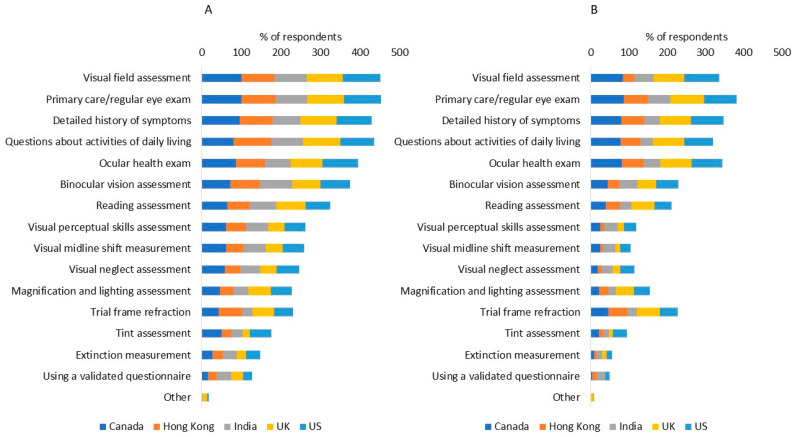
Country-specific percentages of respondents regarding post-stroke visual assessments. (**A**) Assessments PERSONALLY CONSIDERED to be important (**B**) Assessments UNDERTAKEN for post-stroke individuals.

Most participants (>50%) considered several visual assessments to be important for individuals who have had a stroke (Figure 3a). These included routine eye exams (93.1%); ocular health exams (slit lamp examination and dilated fundus evaluation—83%); a detailed case history of symptoms (87.3%); specific questions about activities of daily living (86.2%); assessments of visual field (92.4%) and binocular vision beyond what would be included in primary care (75.4%); and tests involving reading (64.5%), visual perceptual skills (53.6%), visual neglect (52.9%), and visual midline shift (53.3%). This trend was consistent across all countries, as shown in Figure A6 in Appendix A. However, in all cases, few optometrists actually undertook the assessments that they thought important.

**Figure A7:**
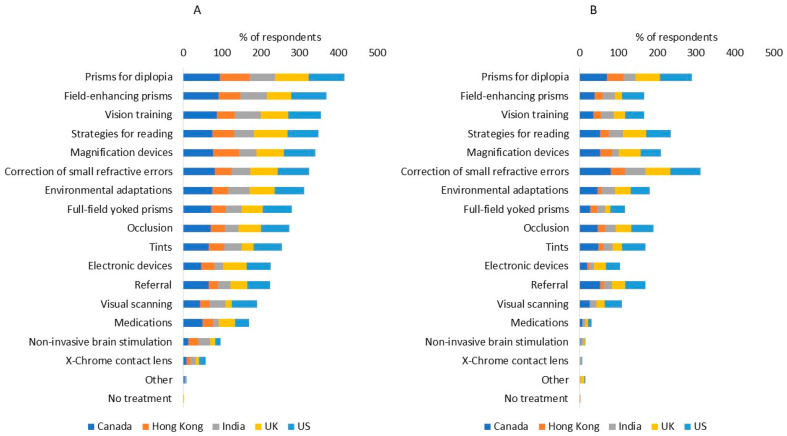
Country-specific percentages of respondents regarding post-stroke visual interventions. (**A**) Interventions that COULD BE RECOMMENDED (**B**) Interventions that were PERSONALLY UNDERTAKEN.

Overall, most (>50%) participants considered that a number of interventions could be recommended for individuals who have had a stroke (Figure 3b). These included prisms for diplopia (87%), field-enhancing prisms (80.8%), vision training (77.2%), strategies for reading (73.6%), magnification devices (73.6%), correction of small refractive errors (72.1%), environmental adaptations (68.5%), full-field yoked prisms (64.1%), occlusion (62.7%), tints (60.5%), electronic devices (51.4%), and referrals to other professionals (50.7%). This trend was consistent across all countries, as shown in Figure A7 in Appendix A. However, in all cases, few optometrists personally undertook the interventions.

**Figure 4:**
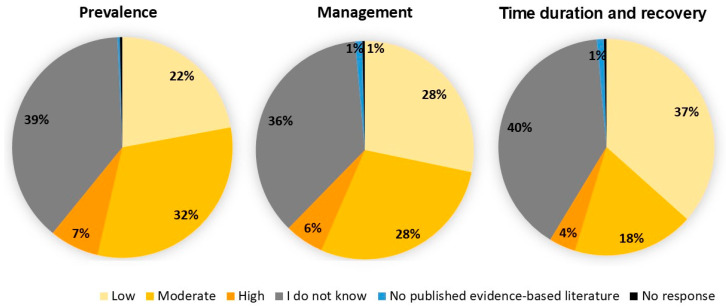
The survey results on the quality of published evidence.

Approximately 39% of the respondents stated that they had no knowledge about the quality of published evidence related to the prevalence, management, time duration, and recovery of post-stroke visual problems (Figure 4). About 22–38% of the respondents believed that the quality of published evidence regarding the prevalence, management, time duration, and recovery of post-stroke visual consequences was either low or nonexistent (Figure 4). However, 61.6% of the participants indicated that enhancing their knowledge on this topic would be helpful in becoming more involved in post-stroke care.

## 4. Discussion

The results of the study show that while a majority of participants are familiar with common visual symptoms [27], visual perceptual difficulties, and visual disorders that occur post-stroke, they are less knowledgeable about some of the less common ones. This reduced awareness was consistent across all countries, with respondents from Hong Kong and India demonstrating less knowledge than those from Canada, the UK, and the US. This highlights a need for generalized and targeted education among eyecare professionals to improve awareness of these less common visual problems.

These findings align with several other studies. For instance, Rowe et al. (2013) reported a significant inequality in care provision for stroke survivors with visual impairments across the UK [14]. Similarly, the present study identified disparities in optometrists’ awareness and management practices across different countries. Rowe et al. also noted variability in how vision screening is conducted, which vision tests are used, and how patients are referred to eye care services. Our study reflects these findings, as although many optometrists recognized the importance of specialized assessments—such as visual field evaluations, binocular vision tests, and perceptual skills testing—many did not actually conduct these assessments in their own practice. This suggests that the variability Rowe et al. identified in the UK extends to other countries as well.

Most participants recognized the importance of specialized assessments that go beyond a primary care examination, such as evaluations of visual field, in-depth binocular vision, reading, visual perceptual skills, visual neglect, and visual midline shift, for individuals who have had a stroke [10,28,29,30]. A study by Smith et al. (2018) also highlighted the importance of comprehensive visual assessments for stroke survivors, including evaluations of eye movement, visual field, and perceptual issues. Smith et al. noted that many visual problems go unrecognized by survivors and are instead identified by caregivers, emphasizing the need for informed and vigilant care providers. This aligns with our finding that many optometrists recognize the importance of these additional assessments but face challenges in conducting them regularly [31]. Additionally, incorporating specific questionnaires into the patient’s eye examination that inquire about their daily activities in detail was considered beneficial. This would help to identify visual disabilities and guide management plans to enhance visual function and improve quality of life post-stroke [32,33].

Management options for post-stroke patients may include ophthalmic prisms, vision therapy (head scanning training, visual space/field awareness training), low vision rehabilitation, assistive devices (optical and electronic magnification, signature guides, field enhancement prisms, environmental and lighting adaptations, and reading strategies), eye drops, occlusion, compensatory head posture, or surgery [34,35]. Deciding on the most suitable management option for each patient requires consideration of their unique needs and consultation with other professionals involved in their care team to ensure that management of vision fits into their personalized individual treatment plan [36,37]. While the surveyed optometrists display awareness of the wide range of possible interventions, few respondents have actually undertaken these assessments or interventions themselves. Another study by Rowe et al. (2013) [14] supports the idea that care provision for post-stroke visual impairments does not occur often. Rowe et al. found that nearly 19% of respondents did not personally test vision, while 22% used screening tools, and only 46% followed designated care pathways for stroke survivors with visual problems. Similarly, in our study, although the optometrists displayed awareness of the necessary assessments and interventions, many cited barriers including a lack of resources, such as adequate coverage or fee [19], and a lack of confidence or experience in conducting these assessments. This reiterates that while the knowledge exists, implementation remains a challenge across different settings, potentially due to inadequate training or a lack of standardized protocols, and the lack of a fee for a specialized assessment results in a lack of experience.

A significant proportion of respondents either indicated unawareness of publications or perceived that the quality of published evidence related to post-stroke visual problems is low. In addition, a significant number of respondents expressed uncertainty about the natural history/duration of recovery of certain disorders. In this respect, for many symptoms, disorders, and perceptual difficulties, they were correct, i.e., the evidence is non-existent or limited for visual problems such as accommodation deficits, difficulty with crowded scenes/places, photophobia, visual midline shift, visual extinction, blurred vision, reading difficulty, object and face recognition, and diplopia. This highlights the need for more research on the natural history of various visual problems, their recovery trajectories, and their impact on different age groups. Specifically, more research is required on such topics as the natural history of these vision problems, vision issues in different age groups of individuals post-stroke (as stroke is not limited to older adults), the impact of stroke type and location on specific visual deficits, and the neural mechanisms underlying vision recovery after a stroke. Although there is evidence regarding the natural history of certain visual problems, such as spatial neglect [38], visual field defects [6,39], partial gaze palsy and forced deviation [7], and visual hallucinations [40,41,42], only 8–30% of the respondents correctly reported this, indicating that the evidence, even when it is present, has not reached the practicing optometrists accurately. Additionally, there is a lack of evidence concerning the management of certain post-stroke visual problems.

Studying the long-term impact of post-stroke vision problems on individuals’ quality of life, including activities of daily living, social participation, and emotional well-being, is essential for assessing the need for and the development of appropriate care pathways. Care pathways have recently been suggested for the UK, but these may not be a good fit in all countries, and the role of optometrists outside hospital systems could be expanded to enhance interdisciplinary care [43].

### Limitations

This study has limitations that should be considered when interpreting the findings. First, despite making multiple attempts to distribute the survey information through various organizations, the response rate was low (0.9% in Canada, 1.5% in Hong Kong, 0.38% in India, 0.21% in the UK, and 0.30% in the US). The low response rate could indicate either a lack of interest in post-stroke rehabilitation among optometrists or uncertainty regarding their knowledge, resulting in hesitancy to participate. The low response rate limits the generalizability of the results, as the respondents may not fully represent the broader optometrist population. Even though all optometrists were asked to respond, those who participated might have a greater interest in post-stroke care and see more stroke patients than the average optometrist, potentially skewing the data toward a more favorable view of optometrists’ readiness and involvement

Second, the survey results show a higher percentage of respondents from academic settings compared to the entire population of optometrists. These individuals could have more access to research and continuing education opportunities compared to optometrists in general practice. If these assumptions hold true, it highlights an even greater need for optometrists to become actively involved and integrated into post-stroke rehabilitation efforts beyond what these results suggest.

Third, the questionnaire used for data collection was self-reported, which introduces the possibility of response bias. Participants might have over- or under-reported their knowledge and practices regarding post-stroke vision care, leading to discrepancies between the reported data and actual clinical practice.

## 5. Conclusions

This manuscript contributes new insights into optometrists’ awareness of, and their readiness to manage, visual disorders in survivors of stroke across multiple countries. It also describes the optometrists’ knowledge gaps and gaps that exist in the literature regarding vision disorders in stroke. Overall, the findings of this study emphasize that optometrists are generally aware of the issues that post-stroke patients experience, as well as many of the assessments and methods of management that are possible. Although they have the basic knowledge to provide these services, fewer are providing these interventions than are aware of them at present. However, the majority of optometrists are willing to be more involved in post-stroke care and believe that optometrists should be members of the post-stroke team [19]. There is a need for more specific education for optometrists, more opportunity in terms of a fee for this more specialized service, and adequate resources. Additionally, more specific education and awareness about post-stroke visual symptoms and disorders among other healthcare professionals, stroke survivors, and their families is required. This study underscores the lack of robust evidence of the natural history and management of certain post-stroke visual deficits, calling for more research in this area to better support clinical practice and improve patient outcomes.

By presenting this information, this manuscript aims to provide evidence for the development of new strategies to better incorporate visual assessment and care into post-stroke care. With some changes, optometrists will be well positioned for this undertaking, and thereby improving patient outcomes and contributing to the overall healthcare system.

By evaluating these factors, this manuscript aims to provide evidence for the development of new strategies to better incorporate optometrists into post-stroke care, improving patient outcomes and contributing to the overall healthcare system.

## Data Availability

The raw data supporting the conclusions of this article will be made available by the authors on request.
